# Kombucha as a Solvent for Chitosan Coatings: A New Strategy to Extend Shelf Life of Red Peppers

**DOI:** 10.3390/ma18071605

**Published:** 2025-04-02

**Authors:** Karolina Stefanowska, Magdalena Woźniak, Anna Sip, Róża Biegańska-Marecik, Renata Dobrucka, Izabela Ratajczak

**Affiliations:** 1 Department of Chemistry, Faculty of Forestry and Wood Technology, Poznan University of Life Sciences, Wojska Polskiego 75, 60625 Poznań, Poland; karolina.stefanowska@up.poznan.pl (K.S.); izabela.ratajczak@up.poznan.pl (I.R.); 2 Department of Biotechnology and Food Microbiology, Faculty of Food Science and Nutrition, Poznan University of Life Sciences, Wojska Polskiego 48, 60627 Poznań, Poland; anna.sip@up.poznan.pl; 3 Department of Food Technology of Plant Origin, Faculty of Food Science and Nutrition, Poznan University of Life Sciences, Wojska Polskiego 31, 60624 Poznań, Poland; roza.marecik@up.poznan.pl; 4 Department of Industrial Products and Packaging Quality, Institute of Quality Science, Poznań University of Economics and Business, al. Niepodległości 10, 61875 Poznań, Poland; renata.dobrucka@ue.poznan.pl

**Keywords:** fruit coating, ascorbic acid, fruit quality, barrier properties

## Abstract

Plastic pollution and environmental degradation necessitate the development of natural, biodegradable food preservation materials. This study examined chitosan-based film-forming solutions using kombucha derived from black tea, lemon balm, and chamomile as natural solvents rich in bioactive compounds. Lemon balm kombucha solutions were used to create chitosan films and coat red peppers. The study assessed the mechanical properties of the films and the effects of chitosan coating on peppers, including texture, ascorbic acid content, sensory attributes, and antioxidant activity. Microbiological tests showed that a chitosan–lemon balm kombucha solution acted against *Escherichia coli*, *Pseudomonas aeruginosa* and *Salmonella enterica*. Lemon balm kombucha had high total phenolic (381.67 µg GAeq/mL) and flavonoid (21.05 µg Qeq/mL) contents. The chitosan film exhibited a tensile strength of 11.08 MPa and an elongation at break of 53.45%. The water vapor transmission rate of the obtained chitosan film was 131.84 g/m^2^·24 h. Coated peppers showed a 32% increase in skin strength and retained 11% more ascorbic acid after 15 days. Sensory evaluation revealed no significant differences from controls. These results highlight lemon balm kombucha as a promising natural solvent for chitosan coatings, which have the potential to extend red pepper shelf life and to support food preservation.

## 1. Introduction

When choosing products in a store, consumers usually assess the quality of vegetables purchased based on their appearance and freshness at the time of purchase. Unfortunately, storing vegetables and fruits on store shelves or transporting them to the store negatively affects the integrity of these products, causing them to brown, change taste and texture and lose nutritional value [1]. Furthermore, studies have shown that about one third of the total production of fruits and vegetables is lost before it reaches the consumer [1]. In order to protect food from spoilage and mechanical damage during transport and storage, packaging is used. However, due to the environmental threat posed by plastic packaging, new solutions are being sought that could successfully replace these while also acting as an environmentally friendly alternative.

An example of such a solution is that of edible coatings, which can protect food products against adverse biological factors, allow for the extension of shelf life [2], limit lipid oxidation [3] and reduce moisture loss from food products [4]. Edible coatings applied to products by spraying or immersion perform a barrier function, protect against gas penetration, moisture migration, aroma changes and the exchange of various soluble substances [5,6]. Various types of matrices are used to produce coatings: polysaccharides such as chitosan, cellulose, starch, pectin [7,8,9]; proteins such as casein, bean proteins, soy proteins [10,11,12]; and lipids or composite materials [13].

Chitosan is a natural polysaccharide obtained from chitin, which in turn is obtained from, among other things, the shells of crustaceans constituting industrial waste. Due to its film-forming properties, biocompatibility, and biodegradability, it is a good matrix for the production of natural packaging or coatings for food products [14]. Literature data show that packaging materials and coatings produced on the basis of chitosan exhibit antibacterial and antifungal activity, which is beneficial from the point of view of protecting packaged food from spoiling too quickly [15,16,17]. Chitosan is a polysaccharide that is insoluble in water, but which dissolves well in acidic substances, e.g., acetic, citric, or lactic acids. Studies have shown that the type of solvent used affects the biological and mechanical properties of the obtained chitosan-based materials [18,19]. Current research focuses on seeking out new solvents for obtaining chitosan materials that, apart from their solvent function, are rich in biologically active substances, and which allow for an increase in, among other things, the microbiological activity of the obtained materials [20,21].

Kombucha is a traditional beverage obtained through the fermentation of tea. This process occurs due to the presence of a symbiotic culture of bacteria and yeast (SCOBY), which consists of acetic acid bacteria (AAB), lactic acid bacteria (LAB), and yeast [22,23]. Kombucha is a rich source of bioactive compounds such as phenolic compounds, organic acids, and vitamins [22]. Due to the presence of bioactive compounds, kombucha is a very effective antioxidant [24,25]. It also exhibits antimicrobial activity against strains such as *Pseudomonas aeruginosa*, *Staphylococcus aureus*, and *Escherichia coli*; research shows that this activity varies depending on the length of the fermentation process or the type of tea used in the process [26].

The aim of this work was to obtain chitosan coatings using a natural solvent that is rich in biologically active substances, i.e., kombucha. Three types of tea were used to prepare kombucha: black tea, lemon balm and chamomile. A kombucha that is characterized by the best biological activity was used to produce a chitosan film and a protective coating, which was then used to cover red bell peppers. The plant material was subjected to qualitative tests to assess the potential possibility of extending the shelf life by covering food products with a film-forming solution of chitosan and kombucha based on lemon balm. The obtained results suggest that lemon balm kombucha can be an effective, natural and novel solvent for chitosan, one that is characterized by the additional benefits that result from the richness of the bioactive ingredients contained in it, and the obtained coating allowed for the maintenance of the product’s high quality during its storage.

## 2. Materials and Methods

### 2.1. Research Methodology of Kombucha, Chitosan–Kombucha Solutions and Films

#### 2.1.1. Preparation of Kombucha

The kombucha was prepared by dissolving 70 g of sucrose (Sigma Aldrich, Darmstadt, Germany) in 1 L of distilled and boiled water, followed by adding 4 g of three tea types: chamomile tea, lemon balm and black tea (Herbapol, Lublin, Poland). After brewing for 10 min, the teas were removed from the solution. Once cooled to room temperature, 100 mL of a previously fermented black tea broth was added as a starter culture. Fermentation proceeded at room temperature for 21 days. For analysis, microorganism cells were removed from the kombucha solutions by centrifugation at 5000× *g* for 10 min, followed by microfiltration with Millex GV membrane filters (Millipore, Burlington, MA, USA).

#### 2.1.2. Preparation of Chitosan–Kombucha Solutions

Chitosan, derived from crab shells purchased from Sigma Aldrich (Darmstadt, Germany), was used to obtain film-forming solutions. Three separate chitosan–kombucha solutions were prepared by dissolving 4 g of chitosan in 400 mL of cell-free kombucha solutions made from chamomile, lemon balm, and black teas. The solutions were stirred mechanically. The symbols of the samples received are explained and presented in Table 1.

#### 2.1.3. Preparation of Chitosan–Kombucha Films

To produce the chitosan–kombucha films, the film-forming solutions obtained as in point 2.1.2. were poured into Petri dishes lined with Teflon and allowed to dry at room temperature for 48 h. Only the film obtained using lemon balm kombucha allowed for further mechanical tests. The obtained chitosan–lemon balm kombucha film was described by the symbol LBF, while the literature data [27] for the chitosan film produced with the commonly used acetic acid was used as a control sample, marked with the symbol CF.

#### 2.1.4. Total Phenolic and Flavonoid Content in Kombucha Solutions

The total phenolic content (TPC) in kombucha solutions was determined using the Folin–Ciocalteu procedure. The kombucha solution (0.1 mL) was mixed with Folin–Ciocalteu reagent (0.25 mL, Sigma Aldrich, Steinheim, Germany), and, after 3 min, with Na_2_CO_3_ solution (3mL, 10%, Avantor Performance Materials, Gliwice, Poland). The absorbances at 765 nm in all solutions were measured by a Cary 300 Bio UV–Visible scanning spectrophotometer (Agilent Technologies, Santa Clara, CA, USA) after incubation for 40 min in the dark and at ambient temperature. Results were expressed as gallic acid equivalent in mL of kombucha solution (mg GAeq/mL). All measurements were made in triplicate.

Measurement of the total flavonoid content (TFC) in kombucha solutions was performed based on the aluminum chloride colorimetric method. The kombucha solution (0.1 mL) was mixed with methanol (0.9 mL) and AlCl_3_ solution (2 mL, 2%, Avantor Performance Materials, Gliwice, Poland). The absorbance at 430 nm was read by a Cary 300 Bio UV–Visible scanning spectrophotometer (Agilent Technologies, Santa Clara, CA, USA) after incubation for 45 min in the dark and at ambient temperature. All results are expressed as µg of quercetin equivalent in mL of kombucha solution (µg Qeq/mL). All measurements were made in triplicate.

#### 2.1.5. Preparation of Indicator Microorganism Cultures for Antibacterial Testing

Bacterial strains stored at −20 °C were gently thawed (at room temperature) and then passaged twice in BHI medium (Oxoid, Basingstoke, UK) with 2% glucose (temperature 30–37 °C, depending on the strain, 24 h). Petri dishes with Mueller–Hilton Agar (Oxoid, Basingstoke, UK) were then inoculated with a suspension of the prepared strains containing 10^6^ cfu/mL. The inoculated plates were left for 15 min to absorb microorganisms on their surface. The strains used for testing were *Bacillus subtilis* (food isolate), *E. coli* (ATCC 10536), *P. aeruginosa* (ATCC 15442), and *S. enterica* (clinical isolate).

#### 2.1.6. Antibacterial Activity of Kombucha–Chitosan Solutions

An amount of 10 µL of tested kombucha–chitosan solution was applied to each of the surfaces of plates inoculated with standardized bacterial suspensions (10^6^ cfu/mL). Plates with applied samples were incubated at 30–37 °C (depending on the strain) for 24 h. After incubation, the antimicrobial activity of the tested samples was assessed. For this purpose, the diameters of the inhibition zones formed around the sites of the applied samples were measured using a computer scanning system (MultiScaneBase v14.02). Results are expressed in millimeters.

#### 2.1.7. Water Vapor Transmission Rate

The water vapor transmission rate (WVTR) of kombucha–chitosan films was evaluated following the ISO 2528:2017 standard [28]. For this measurement, vessels containing 10 g of anhydrous calcium chloride (Avantor Performance Materials, Gliwice, Poland) were covered with the film samples, sealed securely, and placed inside a desiccator. The desiccator maintained a relative humidity (RH) of 65%, which was achieved using a saturated solution of sodium chloride (Avantor Performance Materials, Gliwice, Poland), and a controlled temperature of 23 °C. The vessels were weighed with a precision of 0.001 g. The WVTR was determined using the formula presented as the following formula:

$$WVTR=\frac{m\cdot 24}{A}$$

where WVTR—water vapor transmission rate (g/m^2^·24 h), m (g)—mass increase and A (m^2^)—water vapor transmission area.

#### 2.1.8. Mechanical Properties

The mechanical properties of the prepared films were assessed using an Instron universal testing machine (Model 5965, Instron, Norwood, MA, USA), following the ASTMD882-12 standard [29]. Film samples were cut into strips measuring 1.5 cm by 10 cm and conditioned at 23 °C with 50% relative humidity for at least 48 h. The testing was carried out at a speed of 100 mm/min, evaluating mechanical properties such as tensile strength and elongation at break. The average values were derived from ten samples. Tensile strength (TS) was determined using the following equation:

$$TS\;(MPa)=\frac{F_{max}}{A}$$

where F_max_—the maximum load (N) and A—the cross-sectional area of the initial film strip sample (mm^2^).

Elongation at break (EB) was determined using the following equation:

$$EB\left(\%\right)=\frac{L_{1}-L_{0}}{L_{0}}\cdot 100$$

where L_0_—the initial length of the film strip sample (mm) and L_1_—the final length of the film strip sample at breakage (mm).

### 2.2. Research Methodology of Coated Red Bell Peppers

#### 2.2.1. Plant Material

Red bell peppers were purchased from Lidl (Neckarsulm, Germany). The peppers were selected for uniformity, shape, color, and size, and all fruits with defects were rejected. The fruits were randomly divided into 2 groups of 8 fruits. The fruits of the first group were coated with a chitosan–lemon balm kombucha solution prepared as in point 2.1.2. by dipping them twice in the solution, before being dried at room temperature for 24 h. The second group was a control sample and was not coated. Chitosan–lemon balm kombucha-coated peppers were marked with the symbol LBP, while the control group, which was not coated with the solution, was given the symbol CP. Both the coated and control groups were stored for 15 days at 18 °C, simulating supermarket retail conditions.

#### 2.2.2. Texture Parameter Analysis

The measurement of puncture force and skin elasticity was performed using a TA.XT plus texture analyzer (Stable Micro Systems, Godalming, UK). A P/2 probe and a load cell with a maximum capacity of 5 kg were utilized for this purpose. Measurement parameters were as follows: probe speed of 0.5 mm/s, penetration distance of 6 mm, and a contact force of 50 g.

#### 2.2.3. Ascorbic Acid Determination

The extraction of ascorbic acid from the samples was performed using 1% metaphosphoric acid (Sigma Aldrich, Steinheim, Germany). The content was determined using the UPLC method [30] with an LC Agilent Technologies 1260 Infinity system (Waldbronn, Germany) equipped with a Zorbax SB-C18 column (4.6 mm × 150 mm; 5 µm; Agilent Technologies, Santa Clara, CA, USA) and a DAD detector (Agilent Technologies 1260 Infinity, Waldbronn, Germany). The mobile phase consisted of methanol (phase A) and 0.005 mol/L KH_2_PO_4_ solutions (phase B). A gradient elution was applied as follows: a linear gradient from 5% A to 22% A over 6 min, followed by a return to the initial conditions within 9 min. The flow rate was set at 0.7 mL/min. Detection was performed at a wavelength of 245 nm, and quantification was carried out using the external standard method. The determinations were performed in triplicate for two parallel tests (n = 6).

#### 2.2.4. Sensory Evaluation

Sensory evaluation was conducted by a 10-member evaluation panel in sensory assessment booths specifically prepared for the analysis. A structured 9-point hedonic scale was used, with boundary descriptors ranging from “undesirable” to “highly desirable.” The following sensory quality attributes were assessed: color, aroma, firmness (evaluated by touch), and overall appearance.

#### 2.2.5. Radical Cation Scavenging Activity

Antioxidant activity determined by Trolox equivalent antioxidant activity (TEAC) was evaluated by means of the spectrophotometric method as well as the ABTS^+^ method (2,2-eazinobise(3-ethylbenzothiazoline-6-sulfonic acid). 2,2-Azinobis-(3-ethylbenzothiazoline-6-sulphonic acid) radical monocation scavenging activity was determined following a procedure described by Re et al. [31]. The amount of 0.007 mol/L water ABTS^+^ solution was mixed with 0.002 mol/L potassium persulphate solution at a 1:0.5 ratio. The prepared solution was left in the dark for 12–16 h at room temperature. Prior to measurements, the cation radical solution was diluted with a phosphate buffer (PBS) at pH 7.4. The buffer was added until the solution reached absorbance of 0.700 (±0.020). To determine antioxidant activity, an extract of polyphenolic compounds was prepared from fresh red pepper and LBP-coated red pepper (50 μL) was mixed with 5 mL diluted ABTS^+^ solution and its absorption was measured at 734 nm after 6 min at 30 °C, with a phosphate buffer used as a reference sample. The capacity of free radical scavenging was expressed as micromoles of Trolox per g of sample.

#### 2.2.6. Statistical Analysis

The results are expressed as mean standard deviation (SD). Statistical analyses were performed using Statistica 13.1 software (TIBCO Software Inc., Palo Alto, CA, USA) and Excel 2010 (Microsoft Corporation, Redmond, WA, USA). Analysis of one-way and two-way variance (ANOVA) at *p* < 0.05 followed by the post-hoc Tukey test was applied. The relationships between variables were examined using the Pearson correlation coefficient.

## 3. Results and Discussion

### 3.1. Properties of Three Types of Kombucha and Kombucha–Chitosan Solutions

#### 3.1.1. Total Phenolic (TPC) and Flavonoid (TFC) Content in Kombucha Solutions

Phenolic compounds contribute to the bioactive properties of kombucha, affecting its antioxidant and antimicrobial activities, among other things. Consequently, the total phenolic content (TPC) and total flavonoid content (TFC) in the three types of kombucha were analyzed, and the results are presented in Table 2.

The obtained data showed that the highest total phenolic content (381.67 µg GAeq/mL) was characterized by kombucha obtained from lemon balm. Additionally, black tea kombucha contained high TPC (377.90 GAeq/mL), though it was statistically lower than in lemon balm kombucha. Literature data indicated that black tea showed a total phenolic content 137.5 µg GAeq/mL, which was lower than the value obtained for kombucha, at 234.1 µg GAeq/mL [32]. These studies also suggested that the value of total phenolic content was dependent on the time of fermentation—the highest value (234.1 µg GAeq/mL) was recorded after a month of fermentation, while over time this value decreased and was, after 9 months, 80.8 µg GAeq/mL [32]. A higher TPC (420 µg GAeq/mL) value for kombucha based on black tea was obtained in studies conducted by La Torre et al. [32], who carried out the fermentation process for 7 days. This is consistent with the conclusion that the length of fermentation significantly affects the total phenolic content, and that the TPC value decreased with the length of the fermentation process. Similar results have also been obtained by Shahbazi et al. [33], who examined the TPC in kombucha with the addition of plants with medicinal properties such as cinnamon or thyme, their obtained results also indicate a decrease in the TPC over time, which could be related with the acidity of the samples and the presence of enzymes. Moreover, studies have also shown that total phenolic content varies depending on the type of raw material used in the fermentation process [34].

The data obtained for the total flavonoid content exam showed that the highest TFC (21.05 µg Qeq/mL) was observed for kombucha obtained using lemon balm. Literature data showed that the TFC for kombucha based on black tea was 13 µg Qeq/mL, which is a value that is slightly lower than the results obtained in this research (14.28 µg Qeq/mL) [35]. Studies conducted by Shahbazi et al. [33] have shown that the TFC value depended on the raw materials used in the fermentation process but also on the time of its conduct. Unlike for TPC, in the case of TFC, an increase in the value with time could be observed, this is due to the numerous biotransformations that occur during the fermentation process, as well as the stability of some flavonoids such as theaflavin and thearubigen, and the lower stability of e.g., epicatechin, as well as their variable content in the sample depending on the raw material which was used [33].

#### 3.1.2. Antibacterial Properties of Kombucha–Chitosan Solutions

In order to assess the suitability of the three types of kombucha–chitosan solutions for further studies and to indicate which sample is characterized by the highest antibacterial activity, microbiological studies were conducted against selected strains of gram-positive and gram-negative bacteria which can cause food spoilage and additionally be a source of food poisoning. The results are presented in Table 3.

The obtained results show that the broadest spectrum of activity against gram-negative and gram-positive bacteria was demonstrated by chitosan–lemon balm kombucha solution. The LBS sample showed moderate activity against bacteria such as *B. subtilis, E. coli,* including pathogenic *P. aeruginosa* and *S. enterica*. After its application, zones of complete inhibition of the growth of these bacteria were observed. In contrast, other samples showed only static activity against *S. enterica*, i.e., they only slightly reduced the intensity of their growth. Research conducted by Stefanowska et al. [21] showed that the antimicrobial activity of kombucha also depends on the type of raw material used in the fermentation process.

### 3.2. Properties of Lemon Balm Kombucha Films

For further studies, chitosan films were produced using three types of kombucha, the films obtained using black tea and chamomile-based kombucha did not allow for microbiological and mechanical tests.

**Figure 1:**
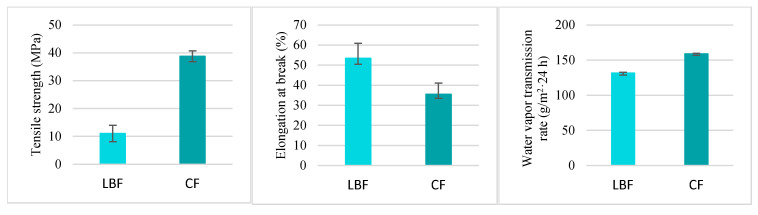
Comparison of mechanical and barrier properties of chitosan–kombucha films versus films produced using acetic acid.

Based on the results of the microbiological tests presented in Section 3.1.2 and the assessment of the suitability of the obtained films for further research, a chitosan film obtained using lemon balm-based kombucha was selected. The results of the tests on mechanical and barrier properties of the obtained film are presented in Figure 1.

The tensile strength (TS) value for the obtained chitosan film was 11.08 MPa, which is twice as high as the value of 4.89 MPa obtained for chitosan films produced using white tea-based kombucha, this value also exceeded the values obtained for chitosan films produced using black tea-based kombucha (3.82 MPa) and coffee-based kombucha (2.77 MPa) [21]. The obtained value is, however, much lower than in the case of the chitosan films produced using the commonly used acetic acid, for which the TS value was 38.80 MPa [27].

In the case of elongation at break (EB), the value obtained for the LBF sample was 53.45%. Literature data showed that EB values obtained for chitosan films produced using kombucha were lower, 24.46% for films based on white tea kombucha and 50.62% for films based on green tea kombucha [21]. A lower EB value (35.5%) was also noted for the film produced with acetic acid as a solvent [27]. Higher values were obtained for chitosan films produced using coffee-based kombucha, 74.5% [21], and for those produced with citric acid, 121.87% [27]. The obtained results and literature data show that the acidic solvent used, or, in the case of kombucha, the mutual proportions of acids present, affected parameters such as TS and EB, which determine the elasticity and strength of the obtained materials [21,27].

The result of the water vapor transmission rate (WVTR) study for the film based on kombucha produced from lemon balm was 131.84 g/m^2^·24 h, which was slightly lower than in the study conducted by Stefanowska et al. [21], where the values obtained for chitosan films based on four different types of kombucha were in the range of 140.8 to 153.2 g/m^2^·24 h. Literature data showed that the WVTR value for chitosan films produced using a traditional solvent, acetic acid, was 159.09 g/m^2^·24 h, which is higher than in the case of the film with kombucha based on lemon balm [27].

### 3.3. Evaluation of the Quality of Red Pepper Coated with Chitosan–Lemon Balm Kombucha Solution

**Figure 2:**
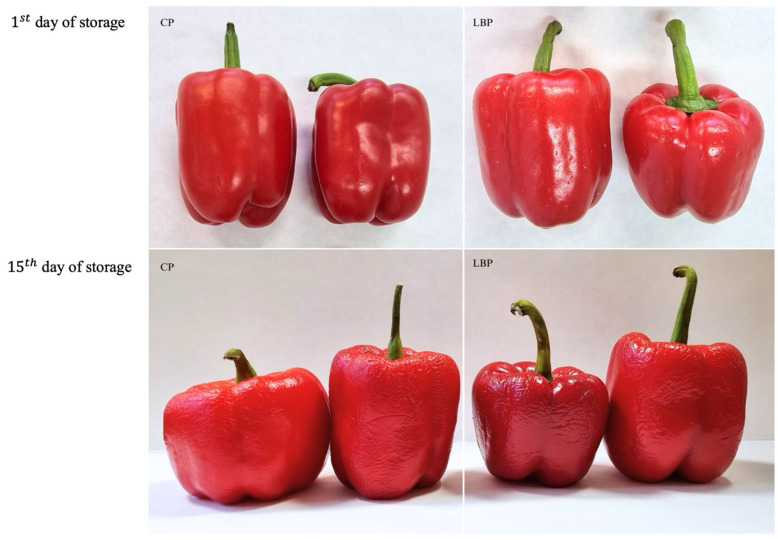
Photographic documentation of the coated red bell peppers and the control sample on days 1 and 15 of storage.

The quality assessment of control peppers (CP) and peppers coated with chitosan–lemon balm kombucha solution (LBS) was conducted based on the measurement of texture parameters, including skin strength and elasticity, sensory evaluation and ascorbic acid, as well as the antioxidant activity of the samples. Photographic documentation of the coated red bell peppers and the control sample on days 1 and 15 of storage is presented in Figure 2.

#### 3.3.1. Texture Parameters

Texture parameters can serve as indicators of changes occurring during the storage of fresh peppers [36]. These changes were described using two parameters: skin strength and skin elasticity. The obtained results are presented in Table 4.

The results indicate that skin strength in LBS peppers showed a significant increase of approximately 32% compared with CP. Up to the 10th day of storage, the applied coating improved skin strength; however, prolonged storage reduced the difference in parameter values to a statistically insignificant level. A similar trend was observed for skin elasticity, which exhibited higher values in coated samples (LBP) up to the 10th day of storage, which increased by approximately 18% on day 1 and around 20% on day 10. Considering that pepper wilting inherently led to a decrease in both parameters, the obtained results indicate a positive effect of the LBS coating on the overall quality of the peppers. Previous studies have indicated that lipid-based coatings derived from mineral oil, as well as protein–wax coatings applied to fruits and vegetables, including peppers, reduce skin damage during distribution and transportation, as well as storage-related injuries [37,38].

#### 3.3.2. Sensory Evaluation

The sensory evaluation of LBS peppers showed comparable scores for the assessed attributes relative to the control sample. This included color desirability, color intensity, and overall appearance; however, for the latter attribute, a significantly higher score was recorded after 15 days of storage. The obtained results of sensory evaluation are presented in Table 5.

The aroma of the evaluated samples was rated more favorably in the uncoated peppers, as the LBS coating altered the typical pepper scent. In the case of the coated samples, the aroma, described as fermentative-sweet-sour, although not perceived as negative, differed from the typical scent of red bell peppers. Nevertheless, the obtained results indicate only minor differences in sensory quality between the LBP samples and the control group. Given the improvement in other quality parameters, such as texture properties and the content of bioactive compounds, this outcome appears fully acceptable, suggesting the potential effectiveness of the LBS coating for application on fruits and vegetables. In the study conducted by Nasrin et al. [39], which compared the sensory quality of red pepper coated with 1.5% and 2% chitosan solution to uncoated pepper after 9 days of storage, and similar to the present study, the appearance and overall acceptability of the chitosan-coated samples were significantly higher compared with the control sample.

#### 3.3.3. Ascorbic Acid Content

Peppers are an excellent source of ascorbic acid, with its content in pepper fruits ranging widely from 60 to 470 mg/100 g FW (fresh weight) [40,41,42,43]. However, it should be noted that the ascorbic acid content in peppers can vary depending on several factors, including variety, cultivation conditions, fruit ripeness, and storage conditions [40,41,42,43]. Therefore, it may serve as a key indicator of pepper quality. The obtained results of the ascorbic acid content analysis are presented in the Table 6.

In the conducted study, the ascorbic acid content in the CP sample was 131.7 mg/100 g FW, while in LBP it was approximately 5% higher. Lemon balm contains around 70 mg/100 g FW of ascorbic acid, therefore, lemon balm kombucha may also contain ascorbic acid, which could contribute to this difference [37]. During storage, the ascorbic acid content fluctuated in both samples, likely due to the natural variability of the pepper material. However, after 15 days of storage, the final ascorbic acid content in LBP was approximately 11% higher than in CP, a difference that was statistically significant. This suggests a beneficial effect of the applied coating in preserving ascorbic acid in peppers. Nasrin et al. [39] have also reported a beneficial effect of chitosan coating (1.5% and 2% solution) on the retention of ascorbic acid content in red pepper during 9 days of storage at 25 ± 2 °C compared with the uncoated control sample. However, the study demonstrated a decrease in ascorbic acid content after the 9th day of storage in both the control and coated samples. In the present study, the observed increase in ascorbic acid content may result from changes in sample mass during storage and the variability of the tested material.

The obtained results of the antioxidant activity are presented in Table 6. The conducted analyses of antioxidant activity demonstrated significantly higher values in the LBP samples compared with CP on days 1, 5, and 15 of storage. The increase in the analyzed parameter in the coated sample compared with the control was approximately 10% after 1, 5, and 10 days, and 24% after 15 days of storage. Moreover, a significant decrease in antioxidant activity was observed in CP after 15 days of storage compared with day 1, where in LBP, this value remained stable, with no significant decline after 15 days. No significant correlation was found between the antioxidant activity of the analyzed samples and their ascorbic acid content. Nevertheless, the higher value of the measured parameter in the LBP samples could be the result of the antioxidant activity of the coating itself [37]. However, the absence of a decline in this parameter during the storage of coated peppers appeared to be beneficial. Kumar et al. [44] reported a decrease in the antioxidant activity of bell pepper coated with a chitosan–pullulan (50:50) composite edible coating enriched with pomegranate peel extract and stored at 23 ± 3 °C. The reduction was approximately 35% in the coated sample and around 45% in the control sample. In the present study, the decrease in antioxidant activity was not statistically significant in the coated sample, amounting to 3.5%, where in the control sample, it was 14%, representing a statistically significant reduction. The antioxidant activity values of the pepper samples were consistent with literature data for red sweet pepper varieties [45,46].

## 4. Conclusions

This study demonstrates that lemon balm kombucha is an effective natural solvent for chitosan-based coatings, one that increases the shelf life of red bell peppers. In addition, kombucha from lemon balm was found to be a rich source of phenolic compounds and flavonoids. Microbiological studies have shown that the chitosan solution made with lemon balm kombucha was active against strains that are undesirable in food, such as *B. subtilis*, *E. coli*, *P. aeruginosa* and *S. enterica.* The TS test of the chitosan film produced using lemon balm kombucha showed that the value of this parameter is twice as high as the value obtained for films commonly produced with acetic acid. Higher values were also recorded for the tested film in the EB test. However, in the WVTR test, the values obtained were slightly lower than those available in the literature data. Red bell peppers coated with LBS retained more ascorbic acid over time compared with the peppers from the control group, indicating better nutrient preservation. Sensory evaluation showed that the coating did not negatively affect the appearance or overall quality of the peppers. Studies have indicated that lemon balm kombucha can be an effective solvent for chitosan with additional properties resulting from the presence of biologically active substances. These findings highlighted the potential of kombucha-based chitosan coatings as a sustainable alternative for food preservation, reducing the impact of storage on product quality and extending shelf life.

## Figures and Tables

**Table 1:** Symbols of received samples.

Symbols	Kombucha Solution	Symbols	Chitosan–Kombucha Solutions
C	Chamomile tea	CS	Chitosan–chamomile tea kombucha
LB	Lemon balm tea	LBS	Chitosan–lemon balm kombucha
B	Black tea	BS	Chitosan–black tea kombucha

**Table 2:** Total phenolic compounds and flavonoid content.

Symbol	TPC (µg GAeq/mL)	TFC (µg Qeq/mL)
C	135.00 ^c^ ± 0.16	13.76 ^b^ ± 1.68
LB	381.67 ^a^ ± 0.05	21.05 ^a^ ± 0.37
B	377.90 ^b^ ± 0.08	14.28 ^b^ ± 0.11

Values are mean ± standard deviation; the different letters within a given parameter indicate a significant difference at *p* < 0.05 for the one-way ANOVA and Tukey post-hoc test means comparisons.

**Table 3:** Antibacterial activity of kombucha–chitosan solutions.

Symbol	Gram-Positive Strain	Gram-Negative Strains		
	*B. subtilis*	*E. coli*	*P. aeruginosa*	*S. enterica*
CS	12.97 ± 0.15	11.03 ± 0.15	12.06 ± 0.11	st
LBS	13.13 ± 0.15	11.10 ± 0.10	12.00 ± 0.10	11.93 ± 0.06
BS	st	st	10.13 ± 0.11	st

Diameter of the resulting zones of inhibition: 5–10 mm—weak activity; 11–14 mm—moderate activity; >14 mm—strong activity; st means weak static action.

**Table 4:** Texture parameters of red bell pepper coated with chitosan–lemon balm kombucha solution and control pepper during 15 days of storage at 18 °C.

Day of Storage	CP	LBP
**Skin Strength (N)**		
1	8.55 ^a^ ± 0.8	11.3 ^c^ ± 1.2
5	10.3 ^b^ ± 0.9	9.8 ^b^ ± 0.7
10	7.63 ^a^ ± 0.7	9.9 ^b^ ± 1
15	10.5 ^b,c^ ± 0.9	10.7 ^b,c^ ± 0.9
Skin Elasticity * (mm)		
1	3.3 ^a^ ± 0.7	3.9 ^a,b^ ± 0.4
5	4.0 ^a,b^ ± 0.7	4.0 ^a,b^ ± 0.8
10	3.5 ^a^ ± 0.3	4.2 ^b,c^ ± 0.4
15	4.7 ^c,d^ ± 0.6	4.9 ^d^ ± 0.8

* Deflection distance of the sample until skin puncture. Values are mean ± standard deviation; the different letters within a given parameter indicate a significant difference at *p* < 0.05 for the two-way ANOVA and Tukey post-hoc test means comparisons.

**Table 5:** Results of sensory evaluation of red bell pepper coated with chitosan–lemon balm kombucha solution (LBS) and control pepper (CP) during 15 days of storage at 18 °C.

Day of Storage	CP	LBP
**Desirability of Color**		
1	9.8 ^c^ ± 0.4	9.4 ^b,c^ ± 0.8
5	9.8 ^c^ ± 0.4	9.1 ^a,b,c^ ± 1.0
10	8.4 ^a,b^ ± 0.8	9.2 ^a,b,c^ ± 0.8
15	8.0 ^a^ ± 0.8	8.4 ^a,b^ ± 0.9
**Color Intensity**		
1	9.6 ^c^ ± 0.5	9.8 ^c^ ± 0.4
5	9.6 ^c^ ± 0.9	9.6 ^c^ ± 0.5
10	8.8 ^a,b,c^ ± 0.4	9.2 ^b,c^ ± 0.4
15	7.8 ^a^ ± 0.9	8.2 ^a,b^ ± 0.4
**Aroma**		
1	9.2 ^c^ ± 0.8	5.2 ^a,b^ ± 0.7
5	8.6 ^c^ ± 1.0	3.8 ^a^ ± 0.8
10	8.8 ^c^ ± 0.9	5.8 ^b^ ± 0.8
15	8.0 ^c^ ± 1.0	6.0 ^b^ ± 0.9
**Overall Appearance**		
1	9.2 ^e^ ± 0.9	8.8 ^d,e^ ± 0.9
5	7.8 ^c,d,e^ ± 1.0	7.6 ^c,d^ ± 1.0
10	6.0 ^a,b^ ± 1.0	5.4 ^a,b^ ± 0.9
15	4.8 ^a^ ± 0.8	6.6 ^b,c^± 0.8

Values are mean ± standard deviation; the different letters within a given parameter indicate a significant difference at *p* < 0.05 for the two-way ANOVA and Tukey post-hoc test means comparisons.

**Table 6:** Content of ascorbic acid and antioxidant activity of red pepper coated with chitosan–lemon balm kombucha solution (LBS) and control red pepper (CP) during 15 days of storage at 18 °C.

Day of Storage	Ascorbic Acid Content (mg/100/g FM)		Antioxidant Activity (µmol of Trolox/g FM)	
	CP	LBP	CP	LBP
1	131.0 ^b^ ± 1.0	139.0 ^b,c,d^ ± 5.1	10.5 ^c,d^ ± 0.08	11.6 ^e^ ± 0.2
5	102.0 ^a^ ± 2.9	143.0 ^d^ ± 0.4	8.4 ^a^ ± 0.3	9.4 ^b^ ± 0.2
10	133.0 ^b,c^ ± 2.8	140.0 ^c,d^ ± 2.6	9.7 ^b,c^ ± 0.2	10.6 ^c,d^ ± 0.7
15	144.0 ^d^ ± 4.2	160.0 ^e^ ± 1.6	9.0 ^a,b^ ± 0.3	11.2 ^d,e^ ± 0.1

Values are mean ± standard deviation; the different letters within a given parameter indicate a significant difference at *p* < 0.05 for the two-way ANOVA and Tukey post-hoc test means comparisons.

## Data Availability

The original contributions presented in this study are included in the article. Further inquiries can be directed to the corresponding author.

## References

[1] Barman K., Ahmad M.S., Siddiqui M.W. (2015). Factors Affecting the Quality of Fruits and Vegetables: Recent Understandings. Postharvest Biology and Technology of Horticultural Crops.

[2] Abdollahzadeh E., Nematollahi A., Hosseini H. (2021). Composition of Antimicrobial Edible Films and Methods for Assessing Their Antimicrobial Activity: A Review. Trends Food Sci. Technol..

[3] Kazemian-Bazkiaee F., Ebrahimi A., Hosseini S.M., Shojaee-Aliabadi S., Farhoodi M., Rahmatzadeh B., Sheikhi Z. (2020). Evaluating the Protective Effect of Edible Coatings on Lipid Oxidation, Fatty Acid Composition, Aflatoxins Levels of Roasted Peanut Kernels. J. Food Meas. Charact..

[4] De Pilli T. (2020). Development of a Vegetable Oil and Egg Proteins Edible Film to Replace Preservatives and Primary Packaging of Sweet Baked Goods. Food Control.

[5] Kõrge K., Bajić M., Likozar B., Novak U. (2020). Active Chitosan-Chestnut Extract Films Used for Packaging and Storage of Fresh Pasta. Int. J. Food Sci. Technol..

[6] Yousuf B., Qadri O.S., Srivastava A.K. (2018). Recent Developments in Shelf-Life Extension of Fresh-Cut Fruits and Vegetables by Application of Different Edible Coatings: A Review. LWT.

[7] Garcia L.C., Pereira L.M., De Luca Sarantópoulos C.I.G., Hubinger M.D. (2012). Effect of Antimicrobial Starch Edible Coating on Shelf-Life of Fresh Strawberries. Packag. Technol. Sci..

[8] Kumarihami H.M.P.C., Kim Y.H., Kwack Y.B., Kim J., Kim J.G. (2022). Application of Chitosan as Edible Coating to Enhance Storability and Fruit Quality of Kiwifruit: A Review. Sci. Hortic..

[9] Rohasmizah H., Azizah M. (2022). Pectin-Based Edible Coatings and Nanoemulsion for the Preservation of Fruits and Vegetables: A Review. Appl. Food Res..

[10] Moghadam M., Salami M., Mohammadian M., Khodadadi M., Emam-Djomeh Z. (2020). Development of Antioxidant Edible Films Based on Mung Bean Protein Enriched with Pomegranate Peel. Food Hydrocoll..

[11] Mohanty B. (2020). Functionality of Protein-Based Edible Coating-Review. J. Entomol. Zool. Stud..

[12] Yousef A.R., Abd El-Moniem E.A., Sh Mahmoud T.M. (2020). Edible Coating of Soy Protein or Gelatin as a Carrier of Thyme Oil for Maintaining Quality of “Barhee” Dates Fruits During Cold Storage. Plant Arch..

[13] Yousuf B., Sun Y., Wu S. (2022). Lipid and Lipid-Containing Composite Edible Coatings and Films. Food Rev. Int..

[14] Wang H., Ding F., Ma L., Zhang Y. (2021). Edible Films from Chitosan-Gelatin: Physical Properties and Food Packaging Application. Food Biosci..

[15] Amor G., Sabbah M., Caputo L., Idbella M., De Feo V., Porta R., Fechtali T., Mauriello G. (2021). Basil Essential Oil: Composition, Antimicrobial Properties, and Microencapsulation to Produce Active Chitosan Films for Food Packaging. Foods.

[16] Kumar N., Neeraj, Pratibha, Singla M. (2020). Enhancement of Storage Life and Quality Maintenance of Litchi (*Litchi Chinensis* Sonn.) Fruit Using Chitosan:Pullulan Blend Antimicrobial Edible Coating. Int. J. Fruit Sci..

[17] Melo N.F.C.B., de Lima M.A.B., Stamford T.L.M., Galembeck A., Flores M.A.P., de Campos Takaki G.M., da Costa Medeiros J.A., Stamford-Arnaud T.M., Montenegro Stamford T.C. (2020). In Vivo and in Vitro Antifungal Effect of Fungal Chitosan Nanocomposite Edible Coating against Strawberry Phytopathogenic Fungi. Int. J. Food Sci. Technol..

[18] Melro E., Antunes F.E., da Silva G.J., Cruz I., Ramos P.E., Carvalho F., Alves L. (2020). Chitosan Films in Food Applications. Tuning Film Properties by Changing Acidic Dissolution Conditions. Polymers.

[19] Qiao C., Ma X., Wang X., Liu L. (2021). Structure and Properties of Chitosan Films: Effect of the Type of Solvent Acid. LWT.

[20] Adımcılar V., Kalaycıoğlu Z., Akın-Evingür G., Torlak E., Erim F.B. (2023). Comparative Physical, Antioxidant, and Antimicrobial Properties of Films Prepared by Dissolving Chitosan in Bioactive Vinegar Varieties. Int. J. Biol. Macromol..

[21] Stefanowska K., Woźniak M., Majka J., Sip A., Mrówczyńska L., Waśkiewicz A., Kozak W., Dobrucka R., Ratajczak I. (2023). A New Approach to Obtain Chitosan Films—Characteristics of Films Prepared with Tea and Coffee Kombucha as Natural Chitosan Solvents. Ind. Crops Prod..

[22] de Miranda J.F., Ruiz L.F., Silva C.B., Uekane T.M., Silva K.A., Gonzalez A.G.M., Fernandes F.F., Lima A.R. (2022). Kombucha: A Review of Substrates, Regulations, Composition, and Biological Properties. J. Food Sci..

[23] Soares I.F., de Lima M.A., Lucarini M., Durazzo A., Arcanjo D.D.R., Lima S.K.R., da Silva R.A. (2023). The Kombucha Ingestion Benefits on the Intestinal Microbiota. Rend. Lincei Sci. Fis. Nat..

[24] Massoud R., Khosravi K., Jafari-Dastjerdeh R., Naghavi N., Khosravi-Darani K. (2022). All Aspects of Antioxidant Properties of Kombucha Drink. Biointerface Rep. Appl. Chem..

[25] Wang X., Wang D., Wang H., Jiao S., Wu J., Hou Y., Sun J., Yuan J. (2022). Chemical Profile and Antioxidant Capacity of Kombucha Tea by the Pure Cultured Kombucha. LWT.

[26] Vohra B.M., Fazry S., Sairi F., Babul-Airianah O. (2018). Effects of Medium Variation and Fermentation Time on the Antioxidant and Antimicrobial Properties of Kombucha. Malays. J. Fundam. Appl. Sci..

[27] Stefanowska K., Woźniak M., Majka J., Sip A., Mrówczyńska L., Kozak W., Dobrucka R., Ratajczak I. (2023). Chitosan Films with Caffeine and Propolis as Promising and Ecofriendly Packaging Materials. Appl. Sci..

[28] (2017). Sheet Materials—Determination of Water Vapour Transmission Rate (WVTR)—Gravimetric (Dish) Method.

[29] (2012). Standard Test Method for Tensile Properties of Thin Plastic Sheeting.

[30] Howard L.A., Wong A.D., Perry A.K., Klein B.P. (1999). β-Carotene and Ascorbic Acid Retention in Fresh and Processed Vegetables. J. Food Sci..

[31] Re R., Pellegrini N., Proteggente A., Pannala A., Yang M., Rice-Evans C. (1999). Antioxidant Activity Applying an Improved ABTS Radical Cation Decolorization Assay. Free Radic. Biol. Med..

[32] La Torre C., Fazio A., Caputo P., Plastina P., Caroleo M.C., Cannataro R., Cione E. (2021). Effects of Long-Term Storage on Radical Scavenging Properties and Phenolic Content of Kombucha from Black Tea. Molecules.

[33] Shahbazi H., Hashemi Gahruie H., Golmakani M.T., Eskandari M.H., Movahedi M. (2018). Effect of Medicinal Plant Type and Concentration on Physicochemical, Antioxidant, Antimicrobial, and Sensorial Properties of Kombucha. Food Sci. Nutr..

[34] Teixeira Oliveira J., Machado da Costa F., Gonçalvez da Silva T., Dotto Simões G., dos Santos Pereira E., Quevedo da Costa P., Andreazza R., Cavalheiro Schenkel P., Pieniz S. (2023). Green Tea and Kombucha Characterization: Phenolic Composition, Antioxidant Capacity and Enzymatic Inhibition Potential. Food Chem..

[35] Ivanišová E., Meňhartová K., Terentjeva M., Godočíková L., Árvay J., Kačániová M. (2019). Kombucha Tea Beverage: Microbiological Characteristic, Antioxidant Activity, and Phytochemical Composition. Acta Aliment..

[36] Liu L., Wei Y., Shi F., Liu C., Liu X., Ji S. (2015). Intermittent Warming Improves Postharvest Quality of Bell Peppers and Reduces Chilling Injury. Postharvest Biol. Technol..

[37] Kazimierczak R., Hallmann E., Sokołowska O., Rembiałkowska E., Kazimierczak R., Hallmann E., Sokołowska O., Rembiałkowska Sggw E., Nauk Żywieniu Człowieka Konsumpcji W., Żywności Ekologicznej Z. (2011). Zawartość Związków Bioaktywnych w Roślinach Zielarskich z Uprawy Ekologicznej i Konwencjonalnej. J. Res. Appl. Agric. Eng..

[38] Kowalczyk D., Pikula E. (2010). Wpływ Jadalnej Powłoki Białkowo-Woskowej Na Jakość Przechowalniczą Winogron (*Vitis Vinifera* L.). Żywność Nauka Technol. Jakość.

[39] Nasrin T.A.A., Rahman M.A., Islam M.N., Arfin M.S., Akter L. (2018). Effect of Edible Coating on Postharvest Quality of Bell Pepper at Ambient Storage. Bull. Inst. Trop. Agric..

[40] Navarro J.M., Flores P., Garrido C., Martinez V. (2006). Changes in the Contents of Antioxidant Compounds in Pepper Fruits at Different Ripening Stages, as Affected by Salinity. Food Chem..

[41] Chilczuk B., Staszowska-Karkut M., Materska M., Michałojć Z. (2019). Zmiany Zawartości Witaminy C w Owocach Czterech Odmian Papryki-Chłodzonych, Mrożonych i Liofilizowanych w Zależności Od Okresu Przechowywania. Żywność Nauka Technol. Jakość.

[42] Davey M., Van Montagu M., Inze D., Sanmartin M., Kanellis A., Smirnoff N., Benzie I.J.J., Strain J.J., Favell D., Fletcher J. (2000). Plant L-Ascorbic Acid: Chemistry, Function, Metabolism, Bioavailability and Effects of Processing. J. Sci. Food Agric..

[43] Surma-Zadora M., Cieślik E., Grzych-Tuleja E., Bodzioch A. (2011). Próba Znalezienia Współzależności Pomiędzy Zawartością Witaminy C a Barwą. Bromatol. Chem. Toksykol..

[44] Kumar N., Pratibha, Neeraj, Ojha A., Upadhyay A., Singh R., Kumar S. (2021). Effect of Active Chitosan-Pullulan Composite Edible Coating Enrich with Pomegranate Peel Extract on the Storage Quality of Green Bell Pepper. LWT.

[45] Sim K.H., Sil H.Y. (2008). Antioxidant Activities of Red Pepper (*Capsicum Annuum*) Pericarp and Seed Extracts. Int. J. Food Sci. Technol..

[46] Hamed M., Kalita D., Bartolo M.E., Jayanty S.S. (2019). Capsaicinoids, Polyphenols and Antioxidant Activities of *Capsicum Annuum*: Comparative Study of the Effect of Ripening Stage and Cooking Methods. Antioxidants.

